# Feasibility of Primary Tumor Culture Models and Preclinical Prediction Assays for Head and Neck Cancer: A Narrative Review

**DOI:** 10.3390/cancers7030858

**Published:** 2015-08-28

**Authors:** Amy J. C. Dohmen, Justin E. Swartz, Michiel W. M. Van Den Brekel, Stefan M. Willems, René Spijker, Jacques Neefjes, Charlotte L. Zuur

**Affiliations:** 1 Department of Head and Neck Surgery and Oncology, The Netherlands Cancer Institute—Antoni van Leeuwenhoek, Plesmanlaan 121, Amsterdam 1066 CX, The Netherlands; E-Mails: m.vd.brekel@nki.nl (M.W.M.V.D.B.); c.zuur@nki.nl (C.L.Z.); 2 Department of Cell Biology, the Netherlands Cancer Institute—Antoni van Leeuwenhoek, Plesmanlaan 121, Amsterdam 1066 CX, The Netherlands; E-Mail: j.neefjes@nki.nl; 3 Department of Otorhinolaryngology—Head and Neck Surgery, University Medical Center Utrecht, Heidelberglaan 100, Utrecht 3508 GA, The Netherlands; E-Mail: j.e.swartz@umcutrecht.nl; 4 Department of Pathology, University Medical Center Utrecht, Heidelberglaan 100, Utrecht 3508 GA, The Netherlands; E-Mail: s.m.willems-4@umcutrecht.nl; 5 Medical library, Academic Medical Center, Amsterdam 1100 DE, The Netherlands; E-Mail: r.spijker@amc.uva.nl; 6 Dutch Cochrane Centre, Julius Center for Health Sciences and Primary Care, University Medical Center Utrecht, Heidelberglaan 100, Utrecht 3508 GA, The Netherlands

**Keywords:** head neck cancer, primary cell cultures, chemosensitivity, radiosensitivity, personalized therapy

## Abstract

Primary human tumor culture models allow for individualized drug sensitivity testing and are therefore a promising technique to achieve personalized treatment for cancer patients. This would especially be of interest for patients with advanced stage head and neck cancer. They are extensively treated with surgery, usually in combination with high-dose cisplatin chemoradiation. However, adding cisplatin to radiotherapy is associated with an increase in severe acute toxicity, while conferring only a minor overall survival benefit. Hence, there is a strong need for a preclinical model to identify patients that will respond to the intended treatment regimen and to test novel drugs. One of such models is the technique of culturing primary human tumor tissue. This review discusses the feasibility and success rate of existing primary head and neck tumor culturing techniques and their corresponding chemo- and radiosensitivity assays. A comprehensive literature search was performed and success factors for culturing *in vitro* are debated, together with the actual value of these models as preclinical prediction assay for individual patients. With this review, we aim to fill a gap in the understanding of primary culture models from head and neck tumors, with potential importance for other tumor types as well.

## 1. Introduction

Seventy percent of all patients with head and neck squamous cell carcinoma (HNSCC) present with advanced stage disease and are characterized by an overall 5-year survival rate of approximately 35%–60% in the case of surgical treatment with or without chemotherapy (CT) and radiotherapy (RT) [1,2,3]. From around 1980 onward the addition of high-dose cisplatin to RT (CCRT) has become the routine treatment for locally advanced disease [4]. Nevertheless, a meta-analysis of randomized trials in 2009 indicated that there is only a moderate absolute overall survival benefit of 6.5% at 5 years when adding chemotherapy to loco-regional treatment [5]. A subgroup of head and neck cancer (HNC) patients with HPV-positive oropharynx carcinomas usually shows better prognosis following CCRT [6]. A similar analysis in laryngeal cancer patients also described no survival benefit from the addition of chemotherapy to radiotherapy [7]. Moreover, the addition of high-dose cisplatin to RT is accompanied with a substantial increase in grade three or worse toxicity of 52% to 89% [8]. A more personalized patient selection for this treatment should improve the quality-of-life of the non-responding patient population.

More effective and less toxic targeted therapies have not (yet) penetrated in the treatment of patients with HNC. In recent years, only cetuximab has been registered as a radiosensitizer to improve treatment for advanced HNSCC. Literature, however, shows inconclusive results for survival benefit of this treatment compared to CCRT [9,10]. Unfortunately, this leaves CCRT the mainstream of therapy with rather variable individual clinical outcome. It therefore remains a major challenge in HNSCC to develop novel drugs for improved survival and to reveal patients prior to therapy that will actually benefit from the intended treatment regimen. Consequently, there is a strong need for a preclinical model to identify those tumors of patients that will respond to a particular treatment. One of such models is the technique of culturing primary tumor tissue and testing drugs prior to treatment. In order for a culture model to be feasible as a preclinical treatment prediction tool, it should be a short-term culture technique, resembling the patient’s tumor as closely as possible and it should be low in costs. Xenograft mouse models can be used to assess therapy response as well. However, they are in fact long-term assays in which the patient’s tumor cells adjust to the murine environment, leading to genetic drift of the tumor cells. These models are not optimal, expensive and difficult for multiple drug testing. For these reasons, we excluded xenograft mouse models from our literature search. With this review, we aim to study feasibility and success percentages of previously described fresh primary HNSCC culturing techniques and their preclinical chemo- and radiosensitivity assays.

## 2. Materials and Methods

**Figure 1:**
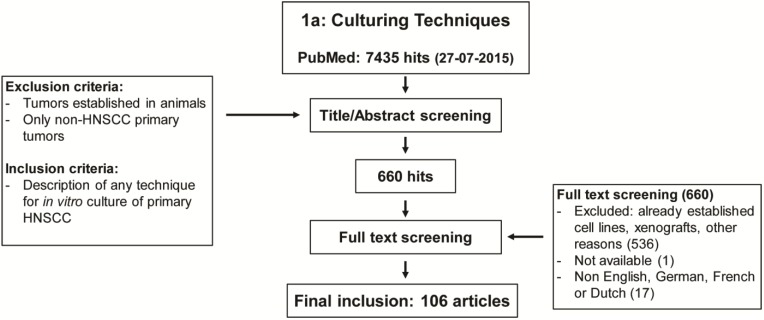

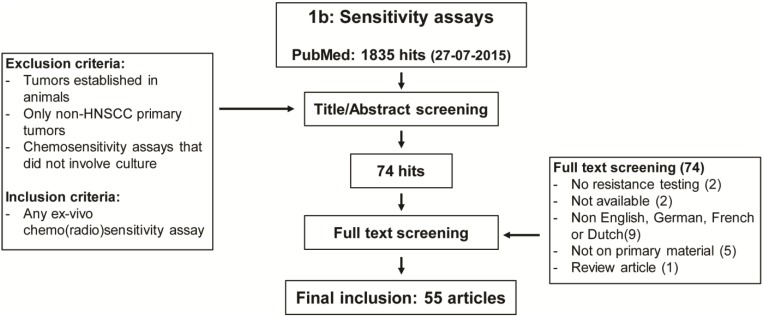
Flow diagram of the systematic review process for the (**a**) search of various culturing techniques used in head and neck cancer; (**b**) search for chemo- and radiosensitivity assays.

A narrative review was performed via a systematic literature search in Pubmed searching for primary HNSCC tumor culturing techniques (research Question 1) and their *in vitro* sensitivity assays with clinical correlation (research Question 2) (Supplementary Materials). We screened titles and abstracts of the identified literature using preformulated criteria (Figure 1a,b). Thereafter, a full text screen of the selected articles was done. Included were studies that described any technique for culturing fresh primary tumor tissue of HNSCC patients, except for techniques involving only cultures using xenografts models. The search includes papers using cell lines. Only papers considering primary tumor tissue to establish fresh cell lines were included. Studies describing the use of purchased or already established cell lines, while not reporting the technique of its establishment, were excluded. Also included were fresh HNSCC culture studies regarding *in vitro versus in vivo* chemosensitivity or radiosensitivity assays. Additionally, references of the included studies were screened and added to the literature list when relevant. Final selection was based on consensus of all authors.

## 3. Results

An overview of key publications for fresh primary tumor cell culture of HNSCC is presented in Table 1, representing the culturing techniques, and Table 2, showing the clinical correlation.

### 3.1. HNSCC Cell Lines

The first attempts to establish HNSCC monolayer cell lines were performed in the 1950’s on a variety of tumors (sarcoma, leukemia, Hodgkin, myeloma, kidney, breast, lung, pharynx, larynx, rectum, melanoma and ovary) [11]. From the 1980s onward, several groups, including those of Carey and Grenman [12], Rheinwald and Beckett [13], and Easty [14], were among the earliest to be able to establish HNSCC cell lines, achieving success rates of around 30% [12]. Nowadays, a myriad of HNSCC cell lines are available for *in vitro* experiments, as summarized by Carey in 1994 [12], Sacks in 1996 [15] and Lin in 2007 [16]. Also, tumor cell lines from particular patient cases are available, such as very young patients or patients with Fanconi anemia-associated HNSCC [17,18]. It is not exactly known why certain tumors may be cultured indefinitely, while others cannot, although culture success rates have improved by taking biopsies under aseptic conditions from non-necrotic and uninfected tumor areas.

In all studies, HNSCC cell lines were established through the “explant technique”, described extensively in 1994 by Carey [12]. In this technique, fresh tumor specimens were mechanically minced into fragments. Samples may be further dispersed enzymatically using trypsin, DNase, collagenase or a combination thereof [19]. The cell suspension was then placed into a rich culture medium, such as DMEM or RPMI-1640 with additional fetal bovine serum (FBS) and transferred to petri-dishes or culture flasks [20,21,22]. A combination of antibiotics and antimycotics was added to prevent bacterial or fungal overgrowth, and fibroblast overgrowth was managed through selective trypsinisation or cell scraping [12]. The cells were then cultured at 37 °C in an air mixture with 5% CO_2_. When cells grew to confluency, they were passaged. According to Carey, a cell line may be considered established after the 20th passage (e.g., several months, depending on growth rate), as about 15% of tumor cells initially show growth but then stop growing or die. Success percentages of 11%–33% have been described for establishment of cell lines from HNSCC in this fashion [13,23,24]. Recently, Owen *et al.* described higher success rates of 50%. They used fluorescence associated cell sorting to separate fibroblasts from tumor cells. This appeared to be a promising technique to reduce fibroblast overgrowth and to improve the success rate of cell line establishment [25].

Regarding *in vitro* to *in vivo* correlation, no significant difference was found between radiosensitivity of HNSCC cell lines established from 7 patients with recurrent disease after RT, and cell lines derived from 13 patients without prior irradiation. Moreover, two patients with unfavorable clinical response to RT, provided cell lines with good RT response *in vitro* [26]. However, these preclinical assays did not consider fresh tumor specimens but cell regrowth from previously established cell lines up to 22 passages, conditions that may have selected cells with reasonable radiosensitivity.

Unfortunately, while assays using HNSCC cell lines have been proven essential for experiments concerning molecular biology, they seem not useful as a preclinical prediction model for the individual cancer patient. It is critical to establish cells in culture that best resemble the patient’s tumor. This implies that the selection on the fast growing stable cells, surviving under culture conditions, should be prevented. Short tissue cultures, where various cells are still present and not out-selected, would be critical to arrive at patient-relevant culture conditions for the testing of various treatment conditions.

### 3.2. Single Cell Cultures

One essential way of culturing is by starting off with single cell suspensions from tumor biopsies. This is usually done by mechanical and enzymatic digestion. The first part of Table 1 describes studies using this technique.

#### 3.2.1. The Cell-Adhesive Matrix (CAM) Assay

The cell adhesive matrix (CAM) assay is a monolayer culture system developed by Baker *et al.*, that uses a fibronectin and fibrinopeptides coated dish for optimized cell adhesion [27]. Cell growth was stimulated through hormone- and growth factor-supplemented medium. Fresh primary tumor biopsies (melanoma, sarcoma, lung, colon, ovarian and renal) were mechanically and enzymatically digested and plated as single cells. After 24 h of incubation, RT or drugs were administered. After 2 weeks the cultures were fixed for quantification of cell growth and survival. Baker *et al.* successfully cultured 75%–90% of tumors using this technique. The articles reviewed, reaches culture success rates of 60%, within 14–21 days of culturing, in studies with a large number of patients (Table 1) [27].

The CAM assay in HNSCC has only been used to assess radiosensitivity. Brock *et al*. first reported on radiosensitivity using the CAM-assay in 1990, in which 72 of 121 HNSCC patients were evaluable (60% success rate) (Table 2) [28,29]. Radiosensitivity was determined by comparing the cell-covered surface to the total surface of 24-well plates after radiation with 2 Gray (surviving fraction at 2 Gray, SF2). The SF2 was 0.40 in 12 patients with recurrent disease and 0.30 in 60 patients with local tumor control (*p* > 0.05).

**Table 1:** Overview of the various culturing techniques from HNSCC tissue.

Authors, year	Assay	Read-out	Result	Corrected for stroma	Days	Patient	Success
						(n)	(%)
**Single cell cultures after enzyme digest**							
Brock 1990	CAM monolayer	SF2	SF2 0.33 (0.11–0.91)	No	14	121	60
Girinsky 1993	CAM monolayer	SF2, alpha	SF2 0.39 (0.37–0.42), alpha 0.18 (0.13–0.24)	No	14–21	96	60
Girinsky 1994	CAM monolayer	SF2, alpha	SF2 0.39 (0.37–0.41), alpha 0.19 (0.14–0.25)	No	14–21	156	60
Eschwege 1997	CAM monolayer	SF2	SF2 0.41 (0.21–0.88)	-	-	92	-
Mattox 1980a	Soft-agar clonogenic	CE (>20 cells), 3-Th	CE 0.006 (0.001–0.08)	No	10–14	36	64
Mattox 1980b	Soft-agar clonogenic	CE (>20 cells)	CE 0.001–0.19	-	14–21	73	45
Johns 1982	Soft-agar clonogenic	CE (≥30 cells, ≥5 colonies)	CE 0.005	No	7–14	73	49
Mattox 1984	Soft-agar clonogenic	CE (≥6 colonies)	CE -	No	14–21	158	36
Cobleigh 1984	Soft-agar clonogenic	CE (≥30 cells, >50 µm, >5 colonies)	Growth observation	No	7–14	51	0
Schiff 1984	Soft-agar and agarose	CE (>20 cells)	CE 0.002–0.08	No	7–21	19	56/90 **
Rofstad 1987	Soft-agar clonogenic	PE (>50 cells), SF2	SF2 ±0.18–0.45. PE 0.6–2.2	No	28–35	4	33
Stausbøl-Grøn 1995	Soft-agar clonogenic	PE (>50 cells, >60 µm), SF2	SF2 0.36 (0.19–0.88). PE 0.02–0.75	Yes	28	15	-
Stausbøl-Grøn 1999a	Soft-agar clonogenic	PE (>60 µm), SF2	SF2 0.50 (0.11–1.00). PE 0.052 (0.005–1.60)	Yes	28	105	70
Stausbøl-Grøn 1999b	Soft-agar clonogenic	PE (>50 cells/>60 µm), SF2	SF2 0.50 (0.19–1.00). PE 0.043 (0.005–1.03)	Yes	28	105	68
Björk-Eriksson 1998	Soft-agar clonogenic	CE (>50 cells/>60 µm), SF2	SF2 0.48 (0.10–1.00). CE 0.093 (0.002–1.30)	Yes	28	140	74
Björk-Eriksson 2000	Soft-agar clonogenic	CE (>50 cells), SF2	SF2 0.40 (0.10–1.00). CE -	Yes	28	156	70
Dollner 2004a	Colony forming (flavin free)	CE (> 16 cells); C100		Yes	4	13	92
Dollner 2004b	Colony forming (flavin free)	CE (> 16 cells); C100		Yes	4	19	89
Dollner 2006a	Colony forming (flavin free)	CE (> 16 cells); C100		Yes	4	13	-
Dollner 2006b	Colony forming (flavin free)	CE (> 16 cells); C100		Yes	4	12	-
**Histocultures**							
Robbins 1994	HDRA	3-Th	Sensitivity: ≥84% IR	Yes	3–15	26	88
Singh 2002	HDRA	MTT, DNA	Sensitivity: >30% IR	No	2	42	98
Ariyoshi 2003	HDRA	MTT	Sensitivity: >40%–60% IR, depending on drug	No	7	19	100
Hasegawa 2007	HDRA	MTT	Sensitivity: >40%–60% IR	No	7	49	-
Pathak 2007	HDRA	MTT	Sensitivity: >50% IR	No	8	57	91
Gerlach 2014	Slice culture on membrane	IHC	Cytotoxic effect	No	5 h–7 days	12	-
Heimdal 2000a	Fragment spheroids	IHC	Viability, cytokine	No	10–28	18	90
Kross 2005	Fragment spheroids	ELISA, IHC	IL-6, MCP-1, TNF-α *	Yes/No	>7	31	-
Kross 2008	Fragment spheroids	ELISA	IL-6, MCP-1 *	Yes/No	10–28	65	-
Lim 2011	Squamospheres	Tumor differentiation, stemcell traits	PCR, IHC, FACS, xenograft	No	>14	47	6
Lim 2012	Squamospheres	Tumor differentiation, stemcell traits	PCR, IHC, FACS, western blot, xenograft	No	>14	-	-

CAM = cell adhesive matrix; ELISA = enzyme-linked immuno sorbent assay; HDRA = histoculture drug response assay; IR = inhibition rate; SF2 = surviving fraction at 2 Gray; PCR = polymerase chain reaction; CE or PE = cloning or plating efficiency; FACS = fluorescence-activated cell sorting; C100 = complete suppression of colony formation; * cytokines and chemokine; MTT = yellow tetrazole is reduced to purple formazan in living cells; ** 56% soft-agar, 90% agarose; IHC = immunohistochemistry.

**Table 2:** Overview of the various assays and their chemo- and radiosensitivity correlations.

Authors, year	Assay	*In vitro* treatment	*In vivo* treatment	Read-out	Correlation	Outcome correlation	FU (months)
**Single cell cultures after enzyme digest**							
Brock 1990	CAM monolayer	RT	Post-op RT	SF2	Yes	Local control. SF2: recurrent 0.40 (n = 12), not yet recurred 0.30 (n = 60). Not significant.	24
Girinsky 1993	CAM monolayer	RT	70% RT, 30% post-op RT	SF2, alpha	Yes	Local control: alpha value. Not for survival	15 (1–29)
Girinsky 1994	CAM monolayer	RT	62% RT, 38% post-op RT	SF2, alpha	Yes	Local control: alpha value	24 (9–47)
Eschwege 1997	CAM monolayer	RT	RT	SF2	No	Local control, survival	68 (45–80)
Mattox 1980a	Soft-agar clonogenic	CT	-	CE, 3-Th	-	-	-
Mattox 1980b	Soft-agar clonogenic	CT	-	CE	Yes	Early mortality: CE > 0.02%	-
Johns 1982	Soft-agar clonogenic	CT	-	CE	Yes	Stage, N-class and survival: high CE (n = 29)	-
Mattox 1984	Soft-agar clonogenic	CT	-	CE	No	No correlation positive culture with stage, N-class, recurrence. No difference in survival for high (>0.02%) and low (<0.02%) CE	24
Cobleigh 1984	Soft-agar clonogenic	-	-	CE	-	-	-
Schiff 1984	Soft-agar and agarose	-	-	CE	-	-	-
Rofstad 1987	Soft-agar clonogenic	RT	-	PE, SF2	-	-	-
Stausbøl-Grøn 1995	Soft-agar clonogenic	RT	RT	PE, SF2	-	-	-
Stausbøl-Grøn 1999a	Soft-agar clonogenic	RT	-	PE, SF2	No	Overall/tumor SF2 were not correlated with T/N and stage	-
Stausbøl-Grøn 1999b	Soft-agar clonogenic	RT	RT	PE, SF2	No	Overall/tumor SF2 and PE did not predict local-regional control (n = 38)	42 (16–70)
Björk-Eriksson 1998	Soft-agar clonogenic	RT	-	CE, SF2	No	SF2 did not correlate with tumor grade, T/N class	
Björk-Eriksson 2000	Soft-agar clonogenic	RT	RT/CT/Surgery	CE, SF2	Yes	Tumor SF2 (0.40) prognostic for local control, not for overall survival. SF2: recurrent 0.53 (n = 14), not yet recurrent 0.38 (n = 70)	25 (7–65)
Dollner 2004a	Colony forming (flavin free)	CT	-	CE, C100	-	-	-
Dollner 2004b	Colony forming (flavin free)	CT	-	CE, C100	-	-	-
Dollner 2006a	Colony forming (flavin free)	CT	-	CE, C100	-	-	-
Dollner 2006b	Colony forming (flavin free)	CT	-	CE, C100	-	-	-
**Histocultures**							
Robbins 1994	HDRA	CT	CT	3-Th	Yes	Clinical response. PPV 83%, NPV 64%. Sensitivity 71%, specificity 78%	-
Singh 2002	HDRA	CT	Surgery/(C)RT/CT	MTT, DNA	Yes	Clinical response. Chemosensitivity is a significant prognostic variable for 2 year cause specific survival	30
Ariyoshi 2003	HDRA	CT	CT and CRT	MTT	Yes	Clinical response. CRT: PPV 87%, NPV 50%. Sensitivity 87%, specificity 50% (patients received RT, *in vitro* no RT)	-
"	"					CT: PPV 90%, NPV 100%. Sensitivity 100%, specificity 67%	
Hasegawa 2007	HDRA	CT	CT then surgery	MTT	Yes	Clinical response. PPV 77%, NPV 80%. Sensitivity 91%, specificity 57%.	> 4 weeks
"	"					Significant correlation between cisplatin sensitivity *in vitro* (50% cut-off) and clinical response. No correlation for 5-FU.	
Pathak 2007	HDRA	CT	Surgery/(C)RT/CT	MTT	Yes	Clinical response. PPV 69%, NPV 80%. Sensitivity 79%, specificity 71%.	2 weeks
"	"					Significant correlation between *in vitro* chemosensitivity and clinical response	
Gerlach 2014	Slice culture on membrane	CT	-	IHC	-	-	-
Heimdal 2000a	Fragment spheroids	-	-	IHC	-	-	-
Kross 2005	Fragment spheroids	-	-	ELISA, IHC	-	-	-
Kross 2008	Fragment spheroids	-	-	ELISA	Yes	Increased IL-6 levels predict recurrence and survival	30
Lim 2011	Squamospheres	CT	-	Differentiation	-	-	-
Lim 2012	Squamospheres	CT	-	Differentiation	-	-	-

CAM = cell adhesive matrix; C100 = complete suppression of colony formation; HDRA = histoculture drug response assay; MTT = a yellow tetrazole, is reduced to purple formazan in living cells; RT = radiotherapy/irradiation; ELISA = enzyme-linked immuno sorbent assay; CT = chemotherapy; IHC = immunohistochemistry; SF2 = surviving fraction at 2 Gray; PPV = positive predictive value; CE or PE = cloning or plating efficiency; NPV = negative predictive value.

In 1994, Girinsky *et al.* described the CAM assay in 156 HNSCC biopsies. SF2 data were available for 76 HNSCC patients [30,31]. SF2 values were not predictive for long-term local control (cut-off 0.50; 66% *versus* 63%). On the other hand, a significantly higher local control rate (*p* = 0.04) was obtained for patients with higher alpha values (which illustrates the rate of cell kill by a single dose of RT; cut-off 0.07 Gy^−1^; 69% *versus* 38% at 2 years).

The third group to work with the CAM assay was Eschwege *et al.* [32]. They studied 92 HNSCC patients with mainly oropharyngeal carcinomas treated with RT and found both SF2 and alpha value not to be prognostic factors for local control and overall survival.

#### 3.2.2. Soft-Agar Clonogenic Assays

Clonogenic assays, in which single tumor cells were cultured on agar-coated plates, were first described by Puck and Marcus on HeLa cervical tumors [33,34]. In 1977, Salmon and Hamburger utilized an adaptation of this technique as an *in vitro* clonogenic assay of anticancer drugs on tumor cells (myeloma, lymphoma, leukemia, lung, ovary, melanoma and neuroblastoma) [35]. Later, it was used for human pancreatic and colon tumor cells grown in immune-suppressed mice, popularized by Courtenay and Mills and referred to as the Courtenay-Mills clonogenic assay [36]. The main feature of this agar method is its selection for stem cells or transformed cells [37,38]. Although agar cultures also support benign tumors and anchorage-dependent cells, if supplemented with high serum levels or transforming growth factors, soft-agar is still a broadly accepted method for tumor cell selection based on their anchorage-independent growth behavior.

The successful use of the Courtenay-Mills soft-agar clonogenic assay with biopsies of HNSCC was first described by Mattox and Von Hoff [39,40,41], Johns [37] and Schiff [42] (Table 1). Primary HNSCC samples were washed, minced with scalpels and further disaggregated, as in the “explant” technique described by Carey [12]. The cell suspensions were placed in culture plates covered with a feeding layer containing agar, culture medium, FBS and a variety of other nutrients, and then incubated with a chemotherapeutic drug for one hour [39]. After that, the cells were washed, plated and incubated, along with untreated controls. After 7–21 days, the cultures can be evaluated for colony formation (clumps of more than 20–40 cells). Plating efficiency (number of colonies compared to number of plated cells) was generally low, around 0.005, meaning only 1 in 200 cells will grow out as a colony. Cultures were regarded successful if six or more colonies form in untreated control plates [37,39,40,41]. The survival fraction was calculated from the amount of colonies formed in treated, compared to untreated plates. Survival rates of 30% or less, compared to untreated controls, were considered an *in vitro* indicator of chemosensitivity [37,39,40,41]. We reviewed several studies using soft-agar clonogenic assays, showing overall success rates of 50% (0%–74%), where colonies of 20 to 50 cells form within a time span of 1–5 weeks [37,39,40,41,42,43,44,45,46,47,48,49]. These studies were done on a reasonably amount of patients (Table 1). More poorly differentiated tumors had higher overall culture success rates than well-differentiated tumors [41,42].

These authors also did *in vivo* correlations with this assay (Table 2). However, chemosensitivity testing was often not possible due to low tumor cell count. Mattox [40] and Johns [37] showed that a higher cloning efficiency (>0.02% and >0.05%) was associated with a higher likelihood of recurrence [37] and early mortality [37,40]. However, a follow-up study of 158 attempted fresh HNSCC cultures did not confirm this correlation [39]. Cobleigh attempted a soft-agar assay on HNSCC in 1984 as well, with no success [43]. Finally, Schiff tried to culture tumors from 19 HNSCC patients [42]. Samples from nine patients were cultured in agar and 10 in agarose. Culture success was higher in agarose-cultured samples (56% *versus* 90%).

With respect to radiosensitivity correlations done with this clonogenic assay, Rofstad, in 1987, studied various tumors (including four head and neck tumors) with a 33% culture success rate [44]. The SF2 differed considerably among individual tumors of the same histological type. In 1995, Stausbøl-Grøn cultured biopsies of 15 HNSCC patients prior to radiotherapy [45]. In 12 tumor biopsies 2%–33% of the colonies were tumor and 83%–100% of the colonies were fibroblasts. The overall SF2 correlated significantly to the fibroblast SF2 but not to tumor cell SF2. In 1999, the same group assessed radiosensitivity in 105 HNSCC patients. Culture was successful in 70%. Data were described from 38 patients who were treated with radiotherapy [46,47]. The majority of the colonies obtained from the biopsies were again fibroblast-marker positive. No significant correlations were found between overall or tumor SF2 and T/N-class and disease stage. Neither tumor cell SF2, overall SF2, nor plating efficiency predicted the locoregional tumor control probability.

Björk-Eriksson determined the intrinsic radiosensitivity of primary HNSCC on data collected over 5 years for 140 patients using a soft-agar clonogenic assay [48]. Care was taken to ensure that only colonies from malignant cells were scored by morphology and staining. Colonies with a radius of more than 60 µm (>50 cells) after 4 weeks of culture were quantified. They reached a culture success rate of 74% (104/140) with a colony-forming efficiency (CFE) of 0.093 and obtained SF2 data from 63% of the patients with a mean of 0.48 (0.10–1.00). Interestingly, these authors observed that approximately 0%–10% of cultured colonies were of a non-malignant cell type. In 2000, the same group reported on 156 previously untreated HNC patients (70% culture success rate, 110/156) and evaluated in 54% (84/156) of the patients the prognostic value of SF2 prospectively [49]. Eighty-four patients were mainly treated with neoadjuvant chemotherapy plus radiotherapy, with or without final surgery. For prognostic analyses, patients were divided in radioresistant (SF2 > 0.40) and radiosensitive (SF2 < 0.40) tumors. After multivariate analysis, tumor SF2 was found an independent prognostic factor for local control (*p* = 0.036), but not for overall survival (*p* = 0.20).

Dollner used a colony forming assay without soft-agar, in a 96-well plate format. In 2000, they used monochromatic light sources to avoid flavin-mediated photo-oxidative effects (termed “Flavino-assay”) especially during chemosensitivity testing. Fresh tumor biopsies were digested and after 3 days of exposure to various drugs adherent colonies were fixed and counted to determine the IC-50 [50,51]. The overall chemoresponse was dominated by stromal cell multidrug resistance [52,53,54]. Stromal cells were resistant to drug combinations in 98% of the experiments, whereas epithelial colonies were sensitive to cisplatin/5-FU in 16%, to carboplatin/5-FU in 8.3%, to cisplatin/docetaxel in 33% and to carboplatin/docetaxel in 8.3%. In 2010, the assay was correlated to clinical outcome in 18 cultures receiving neoadjuvant TPF (docetaxel, cisplatin and 5-FU) prior to RT [55]. Twelve tumors could be successfully cultured (66.7%) The *in vivo* tumor response to induction chemotherapy was correctly predicted by the tumor culture assay in 10 patients (83.3%). However, an *in vitro* prediction of clinical tumor response to the complete treatment regimen was disregarded [55]. These data were only published in a meeting abstract; the full article was not published and thus not fully evaluable. In recent years, this group has used this assay to test chemotherapy response to several drugs *in vitro* [56]. However, no reports have been published reporting a proper correlation between predicted outcome based on the Flavino assay and the actual patient outcome in the clinic.

### 3.3. Histocultures

Another way of culturing is to leave tumor tissue intact by using only mechanical mincing. This maintains the normal and (largely) unaffected tumor-tumor environment interactions as occurring *in vivo*. The second part of Table 1 depicts studies using this technique.

#### 3.3.1. The Histoculture Drug Response Assay (HDRA)

In an effort to preserve the 3-dimensional (3D) histological structure of the tumor, a method was developed to culture (mouse) breast tumor fragments without further dispersal [57], thereby maintaining cell heterogeneity and cell-cell interactions [58]. These models became the cornerstone of the “histoculture drug response assay” (HDRA), further developed by the group of Hoffman for gastric and colorectal cancers [59,60].

Primary tumor material was minced into fragments of about 0.5 mm diameter and placed on 1 × 1 cm collagen sponge gels in a 24-well plate. One mL of RPMI-1640 medium supplemented with FBS was added and the plate was incubated. RPMI medium was selected rather than (D) MEM, for better preservation of phenotypic heterogeneity [61]. For chemosensitivity assessment, drugs were added to the culture medium and cultured for 7 days. Viability was determined using the MTT assay that measures metabolic activity by a spectrophotometer. When the inhibition rate (absorbance in treated, compared to untreated samples) was 50% or more, tumors were regarded as chemosensitive [62].

Robbins and colleagues were the first to describe the HDRA in HNSCC [63]. They investigated inhibition of tumor proliferation by cisplatin using radioactive ^3^H-thymidine incorporation in tumor cells as an endpoint. In a group of 26 patients with HNC (21 SCC, five with other histological types), 23 (88%) specimens were evaluable. The authors described a positive predictive value (PPV) of 83% and a negative predictive value (NPV) of 64% for partial or complete clinical response in patients treated with cisplatin chemoradiation. Singh observed a correlation between *in vitro* chemosensitivity and 2-year cause-specific survival, for cisplatin, 5-FU and both agents [64]. However, the 41 patients included endured various treatment modalities including chemotherapy, surgery and RT. Therefore, no conclusion could be drawn considering chemosensitivity in this study. Another study concerning patients treated for oral cavity SCC showed a PPV of 87% and a NPV of 50% for sensitivity testing with 5-FU, cisplatin, adriamycin, bleomycin and docetaxel [65]. In 2007, Hasegawa *et al.* assessed both primary tumors and lymph node metastases and found a significant correlation between *in vitro* cisplatin sensitivity and clinical response. There was no correlation for 5-FU [62]. Pathak studied a rather homogenous group of oral cavity SCC patients receiving chemotherapy regimens resulting in comparable predictive values [66].

The efficacy and utility of the HDRA as a useful predictor for chemotherapy response in patients is described in a number of studies of various human solid tumors, including gastric and esophageal cancer [67,68], colorectal cancer [59] and ovarian cancer [69,70,71].

Recently, Gerlach and colleagues described an adaptation of the HDRA [72]. In this assay, HNSCC fragments of 12 tumors were sliced with a vibratome or tissue chopper and were placed on membranes, rather than a collagen sponge. Tumor slices were incubated with docetaxel, cisplatin, or no drugs and cultured for 5 h to 7 days in a flavin-free culture medium. The slices were then fixed, embedded in paraffin and examined using Ki-67 (a proliferation marker), caspase-3 staining (an apoptosis marker) and γH2AX (a marker for double-strand DNA breaks). After 7 days of culture, tissue quality was decreased in some tumor slices. Increased apoptosis was observed in the slices exposed to drug, compared to controls. In their publication on this culture method, no correlations to clinical outcomes were done [72]. Recently, more groups have started to generate histocultures of HNSCC to investigate the effect of existing or novel, more targeted drug-based, therapies, such as the PI3K inhibitor LY294002, to investigate the effect of molecular signaling in tumor growth [73,74].

#### 3.3.2. Spheroids, Squamospheres and Organoids

The spheroid culture technique was developed as well to maintain tumor tissue heterogeneity and a 3D architecture (Table 1) [75]. Spheroids would ideally resemble the growth pattern of solid tumors *in vivo* as they are composed of an outer layer of proliferating cells closest to a nutrient and oxygen supply (capillaries) with inner layers of quiescent and -most central- necrotic cells. This was tested with a variety of cell lines [76,77]. Technically, they can either be grown from cells obtained from monolayer cell cultures after trypsinisation or grown from fresh tumor biopsy fragments [16].

In 2000, Heimdal described malignant and benign “fragment spheroids” in a nonadhesive system [78]. HNSCC fragments were cultured on agar-coated culture flasks and after 10–14 days rounded spheroid-like structures were selected for a 2-week co-culture with autologous monocytes derived from peripheral blood samples of the patients. Cytokine IL-6 production of the monocytes was significantly higher in case of direct cell-cell (*i.e.*, tumor-monocyte) contact compared to co-cultures where tumor cells and monocytes were separated by a semi-permeable membrane. In 2005, Kross used the same model to study the cytokine secretion, and to describe the number of epithelial cells (cytokeratin positive), fibroblasts (vimentin-positive) and macrophages (CD68 positive) in both malignant HNSCC and benign spheroids [79]. In malignant spheroids, the proportion of epithelial cells during spheroid formation decreases from 28% to 13%. The density of macrophages (2%) and fibroblasts (13%) did not change. Monocytes secreted more IL-6 when co-cultured with malignant compared to benign spheroids. In 2008 they found increased IL-6 cytokine production *in vitro* to be predictive for recurrence and survival (Table 2) [80].

A few years later, Lim *et al.* described “squamospheres” resulting from culturing mechanically and enzymatically digested biopsies from 47 HNSCC patients [81]. Single cells were incubated for 2–4 weeks to assess sphere forming ability (self-renewal) and other cancer stem cell hallmarks like tumor-initiating capabilities and chemoresistance. A distinction was made between undifferentiated squamospheres (cultured in stem cell medium: serum-free, with N2, B27, EGF and bFGF) and differentiated squamospheres (medium with 10% FBS, without EGF and bFGF). Overall, the success rate of spheroid formation was 6%. Single cells from spheres were assessed for anchorage-independent growth ability as an indicator for cell transformation *in vitro*; undifferentiated cells that maintained sphere forming capability sustained and differentiated cells diminished in agar. In agreement, tumor formation in nude mice was significantly better for undifferentiated cells. This was later confirmed by Pozzi *et al.*, who found better tumorigenicity in sphere forming cancer stem cell (CSC)-enriched cell populations than in unselected tumor cells [82]. To investigate whether HNSCC CSCs can be expanded in adherent cultures without loss of stem cell properties, Lim *et al.* tested different plate coatings [83]. HNSCC-CSCs grew much faster on type IV collagen-coated plates than in suspension. Adherent CSCs expressed stem cell markers, were chemoresistant, produced tumors in mice and showed less spontaneous apoptotic cell death.

Leong *et al.* described the establishment of three cell lines from primary HNSCC grown as spheroids or monolayers. They confirmed the improved chemoresistance of spheroids when treated with 5FU, cisplatin, etoposide or irradiation [84]. Unfortunately, correlation of the *ex vivo* results with the actual clinical outcome was not one of the aims of this study. On the other hand, while sphere formation or sphere formation capability of CSCs, may increase resistance to some drugs, another group has shown in primary HNSCC spheroid cultures that it is also possible to target these CSCs in particular [85].

Until now, only one group described an “organoid culture assay” of HNSCC [86,87]. Although the authors did not use fresh primary tumor, they aimed for 3D *in vitro* tumor growth allowing to form organized and differentiated structures such as those existing in the organism. After full digestion of a xenografted HNSCC in mice, single cell suspension droplets were seeded on a bridge-like filter in a petri-dish. In this model the tissue grows at the air-medium interface, as medium was added just until the bridge. After 4 weeks solid culture nodules were disaggregated again to assess viability of cells by Trypan Blue. Pathologic evaluation of the nodules showed histological characteristics similar to the original human hypopharyngeal carcinoma up to 3 weeks of culturing. After 3 weeks degeneration was seen.

### 3.4. Other Assays

Various other techniques to establish *in vitro* cultures of primary HNSCC were reported, but were only described by a single group and not further popularized. For completeness, these assays are briefly described below.

#### 3.4.1. Flow-Cytometric Analysis

In 1989, Garozzo presented a different model for short-term culturing of HNSCC in which he acknowledged an equal contribution of all cell populations in the progression of neoplastic disease, and referred to Von Hoff stating that HNC are not very likely to grow on agar [88,89]. Surgical HNSCC specimens were disaggregated into cell suspensions and exposed to various drugs for 24 h. The major endpoint was the presence of cell cycle blocks, determined by flow cytometry. Patients were treated with a standardized, undisclosed, regimen of polychemotherapy. Thirteen of the 15 patients showed complete or partial remission. The assay predicted sensitivity to several of the drugs in 11 of these 13 patients (PPV 85%).

#### 3.4.2. Tumor Slices Grown in Test-Tubes

Elprana *et al.* described a culture system where human HNSCC fragments, from one patient, floated freely in test tubes containing medium with or without drugs [90,91]. *In vitro*, the tumor was sensitive to cisplatin and 5-fluorouracil. The patient received a combination of these drugs and experienced complete regression in four months, although long-term outcome was not described.

#### 3.4.3. Microdevices

Recently, the group of Greenman cultured HNSCC samples *in vitro* with a microfluidic device. Medium flowed through the device and was collected after drug or irradiation treatment [92,93,94]. Response to chemotherapy or irradiation is determined by measuring LDH in the effluent. Drug treated samples showed significantly more LDH release than the control groups. No further reports were found that correlated the *in vitro* response to clinical data. This culture technique has been reviewed by Sivagnanam [95].

#### 3.4.4. Micronucleus Assay

Champion *et al.* described an assay that involved establishing a monolayer culture of primary HNSCC tumors and immunohistochemical staining of these cultures after irradiation to identify micronuclei [96]. These micronuclei may be visible in dividing cells and are considered as DNA fragments that cannot be incorporated in daughter cells, due to (radiation) damage. The primary endpoint was the correlation between micronuclei formation and the amount of radiation exposure. After optimizing the assay in cell lines, primary HNSCC specimens were tested. Unfortunately no correlation between assay outcome and clinical outcome could be established.

## 4. Discussion

With this review we aimed to evaluate the most successful *in vitro* culture technique for HNSCC and to discover which model has the best correlation with clinical response. As the chemotherapeutic repertoire increases, a simple and reliable assay to determine the expected patient response becomes critical in making a correct individualized treatment decision.

Monolayer cell line culture is not a proficient method for the use of a preclinical prediction assay. Reasons for this are the long duration of cell line establishment, low culture success rates [12,15] and senescence, the state in which cells no longer divide [13]. Cell line formation is also accompanied with genetic changes like upregulation of oncogenes, and consequently worse clinical outcome [97,98]. Probably for all these reasons, a good clinical correlation was never shown [26].

Short-term fresh tumor cultures, however, do not experience clonal evolution of tumor cell (sub)populations [99]. Worsham, Ragin and Bjerkvig found genetic and molecular cytogenetic resemblance between HNSCC cultures and the primary tumor *in vivo* [100,101,102]. The short duration of culture increases the evaluability of these assays, as these are not influenced by senescence [103,104,105].

Although tumor biopsies are fully digested in the short-term CAM assay, the assay is thought to allow for restored cell-cell contact within the anchored monolayer. It was probably thought that this anchorage was required to establish the predictive value of SF2 for clinical control, however SF2 was not significantly related to outcome in these studies [28,30,31,32]. Only the alpha value (initial slope of radiation curve) had a good clinical correlation with local tumor control in two studies [30,31]. Heppner and colleagues argued that tumor sensitivity to therapeutic agents in a clonal monolayer culture differ to that of *in vivo*-like tissue architectures comprised of heterogeneous cells [106].

Another short-term assay is the soft-agar assay. Von Hoff did a meta-analysis on 54 trials in 1990, using a clonogenic assay, which compared *in vitro* results to clinical outcome in 2300 cases of solid tumors, including a relatively small number of HNSCCs [107]. Overall, they found a 69% true positive rate and a favorable true negative rate of 91%, with a sensitivity and specificity of 79% and 86% respectively, in predicting outcome. We reviewed several studies using soft-agar showing that plating efficiency of HNSCCs is relatively poor. An explanation may be a rather low subpopulation of stem cells in HNSCC. Moreover, solid HNSCC in these studies were fully digested, likely leading to mechanical trauma to the cell. Some authors propose that enzymatic digestion is preferable to maintain viability and growth potential [108]. In addition, the disruption of intercellular attachments may not only irreversibly damage tumor specimens, but may also lead to higher chemosensitivity of cells, not representing the actual *in vivo* sensitivity [109,110,111,112,113]. For example, this is seen in experiments on mouse mammary tumor cell lines; Miller found that chemoresistance to melphalan and 5-fluorouracil was up to a 1000-fold higher in 3D collagen gel structures than in monolayer cell lines [109,110,111]. Unfortunately, research concerning clonogenic assays also failed to systematically show predictive value for individual clinical outcome, probably due to disruption of the tissue. Namely, four studies investigating clinical correlations involving soft-agar HNSCC colony forming assay, did not find any correlation between *in vitro* and *in vivo* response (Table 2) [39,45,46,47]. In two chemosensitivity studies, plating efficiency was associated with tumor stage, N-class and survival [37] and early mortality [40], however not with therapy response. These studies, nevertheless, describe a low number of tumor cells available. Björk performed a radiosensitivity colony forming assay where SF2 was a significant prognostic factor for local control, but not for overall survival [49].

The use of soft-agar should have the advantage of providing support for solid tumor cells, which frequently have difficulties in attaching to the surface of culture dishes. Tumor cells then grow as spherical colonies in agar, while the growth of benign cells such as fibroblast, that require anchorage to a solid substrate, is thought to be reduced [114]. Several groups investigated the impact of stromal cell contamination on culture and treatment sensitivity and concluded that most colonies consisted of fibroblasts. The SF2 is then mainly determined by fibroblast SF2 instead of overall or tumor SF2, and therefore this may contribute as well in not mimicking the correct response *in vivo* [45,48,49,50,51,54,55].

Overall, the number of weeks to culture and the low percentage of evaluable results make the soft-agar clonogenic assay less suitable for use in individual clinical decision making in HNSCC.

In 1994, while other research groups were exploring cultures of fully digested tumor specimens (CAM- and soft-agar assays), Robbins *et al.*, adopted the HDRA mode. This short-term, sponge-supported histoculture of HNSCC tissue fragments does not require enzymatic digest, leaving cell-cell adhesions, 3D character, as well as the tumor heterogeneity intact [60,63]. All cells, benign and malignant, are co-cultured together. This method allows for the formation of cell aggregates with identifiable and distinctive tissue patterns simulating the *in vivo* tumor [57]. This probably explains that, for the first time, very high culture success rates were reached (88%–100%). The hypoxic tumor interior, its low pH and relative inaccessibility to chemotherapeutic agents may be the reason for the high predictive values described for *in vivo* correlations, compared to clonogenic or monolayer assays. In addition, the HDRA needs short-term culturing and will therefore have few genetic alterations when compared to longer-term cultures. Finally, the tumor microenvironment in the HDRA may be of importance for a proper clinical correlation, as the presence of tumor-infiltrating lymphocytes seems to determine clinical outcome in patients with HNSCC [115]. Indeed the HDRA has been confirmed to be a well feasible culture system for fresh HNSCC tissue, as shown by several other research groups with a good correlation to clinical response [62,63,64,65,66]. Only Singh correlated it with clinical outcome, and found that *in vitro* chemosensitivity was a significant prognostic variable for survival [64]. However, these studies tested only chemosensitivity *in vitro*, while patients received chemotherapy often combined with radiotherapy. To improve predictive values and to optimize the clinical relevance for predicting the long-term clinical outcome of HNSCC patients, the HDRA model may be tested not only to determine chemosensitivity, but also radiosensitivity. Furthermore, the clinical follow-up duration or the moment of endpoint determination in the identified studies was not always described or it was short (2 to 4 weeks) [62,66]. This might give an overestimate of the chemosensitivity.

Since 2000, several research groups have focused on growing HNSCC “spheres”, “squamospheres” and “organoids” [79,80,81]. As the term suggests, the investigators aimed to establish a 3D arrangement of tumor cells, forming a sphere or organoid, mimicking solid tumor growth *in vivo*. Therefore, the *in vitro* “3D model” might better mimic drug response *in vivo*. Heimdal and Kross showed the potential importance of immune cells in culture prediction assays using this model. Increased cytokine production in co-culture was significantly higher in direct cell-cell contact between autologous monocytes and tumor [116], and was found to be predictive for a clinical unfavorable prognosis in HNSCC [80]. However, overall, the reviewed studies did not succeed to systematically generate the intended organoid like structures. In addition, growing spheres and organoids as described here is relatively time-consuming. After 2 weeks of spheroid formation, a prediction assay warrants another 2 weeks of incubation. Within the first weeks a decreasing proportion of epithelial cells was seen [80] and a degeneration of histological characteristics [86].

## 5. Conclusions

Within the treatment of HNSCC, there is a strong need for predicting individual clinical outcome prior to therapy, as the overall patient survival rates are relatively low and reliable biomarkers are not available. Moreover, there is a need to test novel drugs before introduction into clinical practice. A preclinical model that closely resembles the *in vivo* situation would be highly valuable. In this review, we observed that the most successful cultures rates and best correlations to clinical response were reported with the HDRA technique. The HDRA assay has the benefit of better representing the tumor and its microenvironment as it does not involve tissue disaggregation, thus maintaining cell heterogeneity, cell-cell interactions and tissue architecture. However, the correlation to clinical outcome of the HDRA technique has been reported only on a small group of patients and should be validated in larger patient cohorts. Within the HDRA technique it is important to correct for stroma cell response. Another outstanding and obvious point is that the clinical treatment should be resembled *in vitro* as closely as possible, including irradiation. This will ultimately determine the success of this culture-based assay for personalized treatment decisions. As it stands, the HDRA technique appears to be the best model to test and identify novel treatment modalities for HNSCC, which is currently specified by a very poor prognosis.

## Supplementary Files

Supplementary File 1
