# Supplementary material for: Feasibility of Primary Tumor Culture Models and Preclinical Prediction Assays for Head and Neck Cancer: A Narrative Review
Source: Cancers (Basel). 2015 Aug 28;7(3):1716–42. doi: 10.3390/cancers7030858 (PMC4586791; doi:10.3390/cancers7030858)
Supplement: Supplementary File 1 [file cancers-07-00858-s001.pdf]

Supplementary Materials

Search strategy for Questions 1 and 2.

Question 1:

(((head[Tiab] OR neck[Tiab] OR tongue[Tiab] OR lip[Tiab] OR cheek[Tiab] OR oral[Tiab] OR oropharyn*[Tiab] OR pharyng*[Tiab] OR pharynx[Tiab] OR larynge*[Tiab] OR larynx[Tiab] OR throat[Tiab] OR glotti*[Tiab] OR nasopharyn*[Tiab] OR hypopharyn*[Tiab] OR "Floor of mouth"[Tiab] OR palate[Tiab] OR retromolar[Tiab] OR gingiva*[Tiab] OR mouth[Tiab] OR ent[Tiab] OR "upper aerodigestive tract"[Tiab] OR UADT[Tiab] OR tonsil*[Tiab]) AND ("squamous cell carcinoma"[Tiab] OR scc[Tiab] OR "carcinoma, squamous cell"[MeSH Terms])) OR (hnscc[Tiab] OR scchn[Tiab] OR "carcinoma, squamous cell of head and neck" [Supplementary Concept])) AND (histoculture[Tiab] OR HDRA[Tiab] OR "cell culture"[Tiab] OR "cells, cultured"[MeSH Terms] OR "single-cell suspension"[Tiab] OR "single-cell suspensions"[Tiab] OR "single cell suspensions"[Tiab] OR "suspension cultures"[Tiab] OR cell-line[Tiab] OR cell-lines[Tiab] OR "cell line"[Tiab] OR "cell lines"[Tiab] OR xenograf*[Tiab] OR "tumor line"[Tiab] OR "tumor lines"[Tiab] OR "primary cell cultures"[Tiab] OR "in vitro model"[Tiab] OR "tissue samples"[Tiab] OR "clonogenic assay"[Tiab] OR tca[Tiab] OR (cells[Tiab] AND culture[Tiab]) OR squamospheres[Tiab] OR "cell culture techniques"[MeSH Terms]).

Question 2:

(((head[Tiab] OR neck[Tiab] OR tongue[Tiab] OR lip[Tiab] OR cheek[Tiab] OR oral[Tiab] OR oropharyn*[Tiab] OR pharyng*[Tiab] OR pharynx[Tiab] OR larynge*[Tiab] OR larynx[Tiab] OR throat[Tiab] OR glotti*[Tiab] OR nasopharyn*[Tiab] OR hypopharyn*[Tiab] OR Floor of mouth[Tiab] OR palate[Tiab] OR retromolar[Tiab] OR gingiva*[Tiab] OR mouth[Tiab] OR ENT[Tiab] OR "upper aerodigestive tract"[Tiab] OR UADT[Tiab] OR tonsi*[Tiab]) AND ("squamous cell carcinoma"[Tiab] OR SCC[Tiab] OR "carcinoma, squamous cell"[MeSH Terms])) OR (HNSCC[Tiab] OR SCCHN[Tiab] OR "Carcinoma, squamous cell of head and neck" [Supplementary Concept])) AND (chemoresistan*[tiab] OR chemosensitivity[Tiab] OR chemotoleran*[tiab] OR radiotoleran*[tiab] OR radioresistan*[tiab] OR radiosensitivity[Tiab] OR chemoradiosensitivity[Tiab] OR "Radiation Tolerance"[Mesh terms] OR ((resistanc*[Tiab] OR susceptibility[Tiab] OR sensitivity[Tiab]) AND (cisplatin [Tiab] OR CDDP [Tiab] OR docetaxel [Tiab] OR taxol [Tiab] OR chemotherapy[Tiab] OR radiotherapy[Tiab]))).
